# Correlation of Phosphorus Adsorption with Chemical Properties of Aluminum-Based Drinking Water Treatment Residuals Collected from Various Parts of the United States

**DOI:** 10.3390/molecules27217194

**Published:** 2022-10-24

**Authors:** Roxana Rahmati, Virinder Sidhu, Rosita Nunez, Rupali Datta, Dibyendu Sarkar

**Affiliations:** 1 Department of Civil, Environmental and Ocean Engineering, Stevens Institute of Technology, Hoboken, NJ 07030, USA; 2 Department of Civil and Mineral Engineering, University of Toronto, Toronto, ON M5S 1A4, Canada; 3 Anisfield School of Business, Ramapo College of New Jersey, Mahwah, NJ 07430, USA; 4 Department of Biological Sciences, Michigan Technological University, Houghton, MI 49931, USA

**Keywords:** water treatment residuals, adsorption, phosphorus, chemical properties, oxalate-extractable aluminum

## Abstract

Over the past several decades, the value of drinking water treatment residuals (WTRs), a byproduct of the coagulation process during water purification, has been recognized in various environmental applications, including sustainable remediation of phosphorus (P)-enriched soils. Aluminum-based WTRs (Al-WTRs) are suitable adsorbent materials for P, which can be obtained and processed inexpensively. However, given their heterogeneous nature, it is essential to identify an easily analyzable chemical property that can predict the capability of Al-WTRs to bind P before soil amendment. To address this issue, thirteen Al-WTRs were collected from various geographical locations around the United States. The non-hazardous nature of the Al-WTRs was ascertained first. Then, their P adsorption capacities were determined, and the chemical properties likely to influence their adsorption capacities were examined. Statistical models were built to identify a single property to best predict the P adsorption capacity of the Al-WTRs. Results show that all investigated Al-WTRs are safe for environmental applications, and oxalate-extractable aluminum is a significant indicator of the P adsorption capacity of Al-WTRs (*p*-value = 0.0002, R^2^ = 0.7). This study is the first to report a simple chemical test that can be easily applied to predict the efficacy of Al-WTRs in binding P before their broadscale land application.

## 1. Introduction

Water treatment residuals are valuable byproducts of the coagulation process in drinking water treatment plants and have been the subject of many studies for more than three decades. Generated as a byproduct of coagulation processes involving iron and aluminum salts, WTRs contain high amounts of Al/Fe hydroxides, making them suitable for many environmental applications. They have been mainly used either as soil amendments to increase their retention capacity and immobilize contaminants, such as arsenic [1], phosphorus [2], vanadium [3], and glyphosate [4], or as adsorbent media to remove phosphorus and heavy metals from stormwater runoff [5,6,7]. They have also been used in aquatic systems for metal immobilization [8] and wastewater for anion (P, Br, F, and Cr) and dye removal [9,10]. Modified WTRs have recently been used to activate peroxymonosulfate and degrade some pesticides and herbicides, such as imidacloprid [11,12] and atrazine [13,14]. They also demonstrated efficiency in activating persulfate to degrade sulfamethoxazole [15].

Increasingly strict potable water standards and population increase have resulted in the production of vast quantities of WTRs worldwide. For instance, in the United States, thousands of metric tons of water treatment residuals are produced daily [16]. They are primarily disposed of via discharge into water bodies, sewer systems, and lagoons or by landfilling [17]. However, all these disposal methods are of economic concern due to high disposal costs [18,19]. Therefore, reusing WRTs would help to minimize waste and provide a sustainable approach to environmental remediation.

Al-WTRs are the most common residuals produced in the US because alum (aluminum sulfate) is the most widely used coagulant in the country [20]. Their inherent physicochemical properties make them suitable adsorbent materials, especially for phosphorus removal [16,21,22,23]. Phosphorus often limits nutrients in surface water eutrophication [24,25], which is a major environmental concern. More than half of the lakes in the US suffer from eutrophication [26,27]. Hence, many studies have focused on binding P in soils or on P removal from stormwater using WTRs.

However, there are still some concerns associated with using these materials, as they are heterogeneous, so their properties are likely to vary widely depending on where they are produced. The first concern is whether all Al-WTRs generated in various treatment plants are safe to reuse. The other is whether all WTRs are equally capable of adsorbing P. There is also a need to understand how their chemical properties influence their performance before considering their large-scale field application. However, performing detailed physicochemical characterizations requires expertise and facilities and could be cumbersome. Therefore, identifying an easily measurable chemical property of Al-WTRs that can predict their P adsorption capacity would be of considerable interest to stakeholders. This study is the first to address these concerns by evaluating thirteen Al-WTRs collected from different geographical locations in the US. The purpose of this work was, first, to assess whether WRTs are nonhazardous and, second, to identify a chemical property that can predict whether a given residual could be successfully reused as a soil amendment to bind P for the remediation of P-enriched soils.

## 2. Materials and Methods

### 2.1. Materials

Al-WTRs were obtained from 13 water treatment plants located in different states of the US from the west to east coast, including New Jersey (NJ), Montana (MO), South Dakota (SD), New York (NY), Florida (FL), Oklahoma (OK), Nebraska (NE), Kentucky (KY), Maine (ME), California (CA), North Carolina (NC), Massachusetts (MA), and Connecticut (CT). All the chemicals used were of analytical grade. Potassium dihydrogen phosphate (KH_2_PO_4_) was used to prepare phosphorus stock solution. PIPES disodium salt and potassium chloride (KCl) were used as a buffer and background electrolyte, respectively. Furthermore, sodium hydroxide (NaOH) and hydrochloric acid (HCl) were used for pH adjustment.

### 2.2. Chemical Properties and Toxicity Analysis of Al-WTRs

Al-WTRs were air-dried for three days upon arrival after separating them from water. Then, they were ground and sieved through a 150 µm sieve for the adsorption experiments and measurement of various parameters, including electrical conductivity (EC); pH; organic matter (OM); moisture; and carbon (C), nitrogen (N), and sulfur (S) contents. A toxicity characteristic leaching procedure (TCLP) was conducted using the EPA method 1311 (USEPA, 1992) to assess the leaching potential of hazardous metals using a synthetic landfill leachate solution. The eight Resource Conservation and Recovery Act (RCRA) metals (i.e., Pb, Hg, Ag, Cd, Cr, As, Se, and Ba), plus Zn, Cu, and Ni, were measured. In addition, a synthetic precipitation leaching procedure (SPLP) was performed based on EPA method 1312 [28] to estimate leaching during a normal rainfall event. Total metals analysis was performed using EPA method 3050B [29]. Oxalate-extractable Al (Al_ox_), Fe (Fe_ox_), and P (P_ox_) were measured by adding the oxalate reagent at pH 3 to samples in darkness [30]. Subsequently, the phosphorus saturation index (PSI) was calculated using Equation (1) [31]:

(1) $$\mathrm{PSI}=\frac{P_{\mathrm{ox}}}{{\mathrm{Al}}_{\mathrm{ox}}+{\mathrm{Fe}}_{\mathrm{ox}}}$$

where P_ox_, Al_ox_, and Fe_ox_ are oxalate-extractable P, Al, and Fe, respectively (mol/kg).

Inductively coupled plasma optical emission spectroscopy (ICP-OES, Agilent 5100, Santa Clara, CA, USA) was used for all analyses, and all experiments were performed in triplicate.

### 2.3. Phosphorus Adsorption Experiments

Batch experiments were carried out to determine the adsorption capacity of Al-WTRs. Constant parameters for all experiments were determined based on previous studies [32,33]. Because runoff is either slightly acidic or basic, the initial pH was adjusted to 7 using 0.01 M HCl or 0.01 M NaOH. Adsorption experiments were performed in triplicate by adding 40 mL phosphorus solution at varying concentrations to 0.4 mg Al-WTR in 50 mL tubes. P solution was prepared using KH_2_PO_4_ stock solution. Initial concentrations used in the experiments were 25, 50, and 100 mg/L, with 0.01 M KCl as background electrolyte and 5 mM PIPES as a buffer. Tubes were then placed on a shaker at 200 rpm for 48 h to reach equilibrium. Experiments were performed at constant ambient temperature (20 ± 1 °C). Initial and final pH was measured, and samples were taken at the beginning and the end of each experiment. Then, samples were centrifuged and filtered. Initial and final phosphorus concentrations were analyzed, and removal efficiencies were calculated using Equation (2):

(2) $$\mathrm{Removal}\;\left(\%\right)=\frac{\left(C_{0}-C_{e}\right)}{C_{0}}\times 100$$

where C_0_ is the initial P concentration (mg/L), and C_e_ is the final P concentration (mg/L). In addition, after each adsorption experiment, the amount of P adsorbed was calculated using Equation (3):

(3) $$q_{e}=\frac{\left(C_{0}-C_{e}\right)V}{m}$$

where q_e_ is the amount of P adsorbed (mg/g), m is the mass of adsorbent (g), and V is the volume of the solution (L). Then, adsorption isotherms were constructed for each sample using the Langmuir isotherm demonstrated in Equation (4):

(4) $$q_{e}=\frac{q_{m}C_{e}K}{1+{\mathrm{KC}}_{e}}$$

where q_m_ (mg/g) is the maximum adsorption capacity, and K is an empirical constant related to entropy. The equilibrium parameter (R_L_) was also calculated using Equation (5) [34,35] to evaluate the suitability of the Langmuir isotherm in this study.

(5) $$R_{L}=\frac{1}{1+{\mathrm{KC}}_{0}}$$

Data analyses were performed using JMP14. Simple linear regression and multiple linear regression were performed to estimate the adsorption capacity based on chemical characteristics of Al-WTRs. The significance level was set at *p* ≤ 0.05.

## 3. Results and Discussion

### 3.1. Metal Concentrations and Toxicity Analysis of Al-WTRs

Total metal concentrations in Al-WTRs are presented in Table S1. The metal content of the various Al-WTRs varied significantly, and Al and Fe concentrations in all samples were much higher than those of other elements. In addition, the Al content in most of the samples was higher than that of Fe, which was expected, given that alum was the primary coagulant in the drinking water treatment processes that generated these residuals.

Although some RCRA-8 metals (e.g., Pb, Cd, As, Cr) were detected in the TCLP leachates, their concentrations were much lower than the USEPA prescribed limits, as shown in Table 1. Concentrations of some elements (Se and Ag) were below detection limits in all samples. Cu, Zn, and Ni were present in very small amounts. These results show that Al-WTRs are nonhazardous materials and can be safely reused for various applications. The non-hazardous nature of WTRs has been confirmed in many other studies, conducted in various countries [7,8,16,35,36]. Because one of the reuse pathways of Al-WTRs is their land application, SPLP was also conducted to determine whether hazardous metals would leach during rainfall events. Results are presented in Table S2. The concentration of metals leached from Al-WTRs under synthetic precipitation was much lower than that of TCLP. Cd, Cr, Pb, Se, Ag, and Ni were below detection limits in all samples. Among RCRA metals, As and Ba were detected in some samples but at concentrations well below their maximum allowable concentrations. Similar findings were reported in previous research [37,38]. These results validate the hypothesis that the field application of WTRs is safe and does not poses any ecological or human health risks.

### 3.2. Chemical Properties of Al-WTRs

The chemical properties of Al-WTRs are summarized in Table 1. The properties varied widely among the 13 samples. Salinity, measured by EC of Al-WTRs, ranged from 0.003 to 1.72 mS/cm, which is considerably below 4 mS/cm, the permittable EC level in soil for salt-sensitive plants. The EC range was similar to that reported by Bai et al. (2014), who worked with WTRs produced in China [39]. The pH values ranged from slightly acidic (6.03) to alkaline (9.78); however, the majority of the samples had near-neutral pH, with an average of 7.4. The observed range was similar to findings for WTRs reported in many other studies and geographic locations such as China [39], the US [18,31,40], and South Africa [41], possibly because alum coagulation is recommended when the source water has a pH of 5 to 7. Samples with higher pH values, such as those from South Dakota, have a high acid-neutralizing capacity and might be useful for resolution of the issue of acidic soils [18].

Al-WTRs also varied in terms of other chemical properties. Organic matter content varied from 0.11 to 14.31%, C from 3.8 to 17.15%, N from 0.12 to 1.33%, and S from 0.32 to 13.43%. Nitrogen contents of samples collected from Oklahoma and Pennsylvania reported by Dayton and Basta [31] were similar to those observed in this study, ranging from 0.005 to 1.8%. Carbon contents were also similar, ranging from 1.7 to 14.9%.

**Figure 1:**
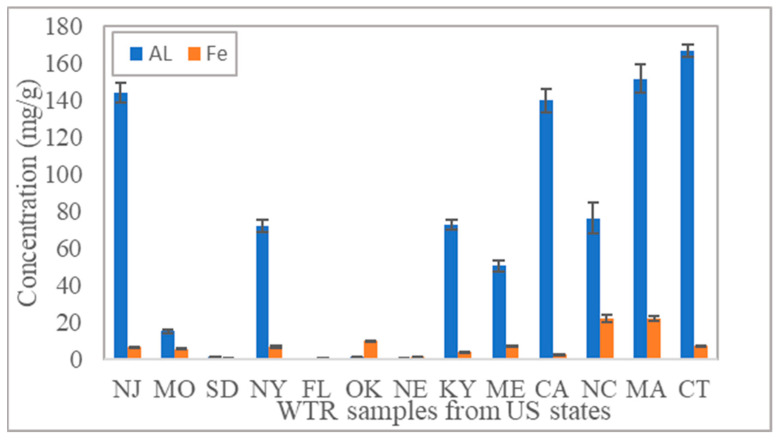
Oxalate-extractable Al and Fe.

Oxalate-extractable Al (Alox) and Fe (Feox) are important chemical properties of WTRs, representing chemical proxies for amorphous Al and Fe oxide/hydroxide surfaces, respectively, that are available for the adsorption of metals and oxyanions [42,43] and have been reported to vary widely from 1 to 160 mg/g in various studies conducted on WTRs collected from various locations [4,8,39,40,41]. In the current study, Alox ranged from 0.27 to 166.54 mg/g, and Feox ranged from 0.48 to 22.2 mg/g (Figure 1).

Because these were Al-based residuals, the concentrations of oxalate-extractable Al were generally considerably higher than those of oxalate-extractable Fe, with a few exceptions, such as the sample collected from Oklahoma, which had a higher Feox content, likely as a result of high Fe concentrations in the source water. High Alox + Feox content indicates that WTRs have highly amorphous Al and Fe surfaces, leading to improved P transport in micropores and high adsorption capacity for P [2]. Hence, samples collected from NJ, CA, MA, and CT were expected to demonstrate higher P adsorption capacity compared to samples collected from SD, FL, OK, and NE (Figure 1).

The phosphorus saturation index (PSI) is a parameter that determines whether reactive Al and Fe surfaces in a solid are saturated with P (PSI > 100%) or whether they are still capable of adsorbing more P (PSI < 100%) [41]. None of the samples were saturated with P (Table 1); however, samples from South Dakota and Nebraska were expected to have lower adsorption capacities, as their PSI was higher than that of other WTRs.

### 3.3. Phosphorus Adsorption on Al-WTRs

Al-WTRs are heterogeneous solids, making their reuse applications challenging, as no two Al-WTRs are the same. To decide whether a given Al-WTR is effective as an amendment in P-rich soils, it is important to identify a parameter that can be relatively easily tested to predict its potential effectiveness as a P-binding agent. The purpose of the adsorption experiments was to determine the maximum adsorption capacities of the 13 investigated Al-WTRs, correlate them with their chemical properties, and identify a single property that can best predict their P adsorption potential.

**Figure 2:**
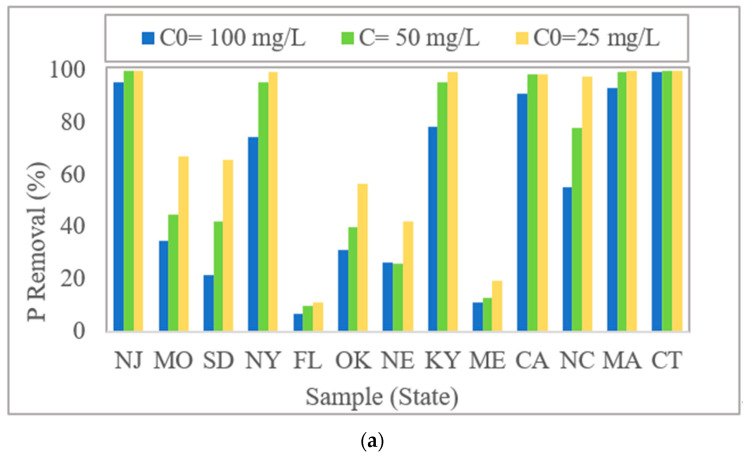

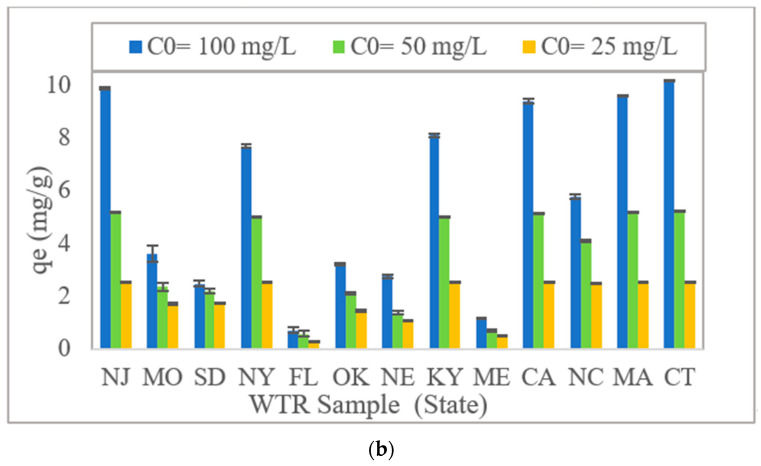
(**a**) Phosphorus removal rate and (**b**) adsorption capacity of Al-WTRs with varying phosphorus concentrations.

Al-WTRs showed a wide range of P removal efficiencies, as shown in Figure 2a. Al-WTR from CT removed more than 99% of P in all three adsorption experiments. Al-WTR from FL showed the lowest removal efficiency of 6.7% for 100 mg/L P solution and 11% for 25 mg/L P solution. The difference can be attributed to the varying chemical properties of the WTRs, particularly oxalate-extractable Al content. Similar observations were reported by other researchers [44,45,46]. More than half of the 13 investigated Al-WTR samples removed more than 97% of phosphorus from the 25 mg/L P solution. This is a significant result, considering that for environmental applications, 25 mg/L P is considered very high. However, such high P concentrations were tested to evaluate the upper threshold of P adsorption capacity of Al-WTRs, similar to the studies by Babatunde et al. [44] and Gysper et al. [47], who worked with 30–150 mg/L P, as well as Bal Krishna et al. [48] and Wang et al. [49], who used even higher concentrations, ranging from 100 to 600 mg/L P. As expected, with increasing initial P concentrations, removal efficiencies decreased, but the range differed from sample to sample. By increasing P concentrations, the P removal rate for some samples decreased significantly, whereas it was not considerably affected for others. For example, P removed using Al-WTR from South Dakota decreased by 67% when the initial P concentration increased from 25 to 100 mg/L in solution, whereas it was only reduced by 4% for Al-WTR from New Jersey. These Al-WTRs differed significantly in their chemical properties, particularly Al_ox_ concentrations (Figure 1).

After each adsorption experiment, the amount adsorbed P was calculated to determine the adsorption capacity (q_e_) corresponding to a given concentration. Results are presented in Figure 2b. Adsorption capacities increased with increasing P concentration in the solution; the degree varied considerably from sample to sample. For the Al-WTR from Massachusetts, the P adsorption capacity in 100 mg/L P solution was 4.5 times that om 25 mg/L P solution; the same ratio was about 2 for Missouri. Researchers suggest that higher adsorption capacity in solutions containing higher P concentration results from an increased diffusion rate due to increased contact [39]. As expected, samples with higher Al_ox_ and Fe_ox_ contents demonstrated higher P adsorption capacities. For the Al-WTR from New Jersey, which had a high Al_ox_ + Fe_ox_ content, q_e_ was three times higher at 100 mg/L P than the Al-WTR from Oklahoma, with low Alox and Feox contents. However, the most important finding is that “all” Al-WTRs were capable of adsorbing P; only their adsorption capacities differed. Similar findings were reported by authors who worked with WTRs from various geographic locations, such as the US, South Africa, Pakistan, and China [31,39,41,50]. Given that all Al-WTRs have a certain P adsorption capacity, regardless of where they are generated in the world, supports our hypothesis that there is a single property (or two) that primarily controls P adsorption in Al-WTRs. In other words, there should be an analyzable chemical parameter that could significantly predict the P adsorption capacity of Al-WTRs.

Adsorption isotherms were constructed for all 13 Al-WTRs. The isotherms were S-shaped and best fit the Langmuir model, as determined based on their high R^2^ values. This observation was similar to that reported by other authors [32,39,44,50,51]. The equilibrium parameter (RL) provided further evidence of the suitability of the Langmuir isotherm model [10]. The value of this dimensionless constant was between 0 and 1 for all Al-WTRs investigated in the study, indicating that this isotherm was favorable.

The phosphorus adsorption mechanism on Al-WTRs is well-established in the literature and was not the focus of this study. The purpose of the adsorption experiments conducted herein was to determine the maximum adsorption capacity of each Al-WTR and to identify a single chemical property that can predict their adsorption potentials. The maximum adsorption capacity (q_m_) for each Al-WTR was calculated based on Langmuir fit, as presented in Table 2. There was a high degree of variability in the maximum adsorption capacities of Al-WTRs, ranging from 1.2 to 28.7 mg/g. Similar ranges were reported in other studies [31,39].

The process of P adsorption on the Al-WTRs was biphasic. The reaction was fast at the beginning, with most P removed from the solution in a matter of minutes. Ligand exchange is the dominant mechanism in this phase, whereas over an extended period, intraparticle diffusion plays a more dominant role in P removal, resulting in a second, slower phase [10,39,46,52,53,54,55]. The ligand exchange reaction is presented in Equation (6) [53], whereby the adsorption of H_2_PO_4_^−^ on Al-WTRs is accompanied by the production of hydroxide ions, subsequently increasing the pH of the solution.

(6) $$\equiv 2\mathrm{Al}-\mathrm{OH}+H_{2}{\mathrm{PO}}_{4}^{-}↔\;{\left(\equiv \mathrm{Al}\right)}_{2}{\mathrm{PO}}_{4}+H_{2}O+{\mathrm{OH}}^{-}$$

The final pH of each experiment was measured, as presented in Figure S1. In most cases, pH increased after the experiment, indirectly supporting the ligand exchange mechanism. In addition, higher initial P concentrations resulted in higher final pH values. When the P concentration increases, the reaction in Equation (6) shifts to the right, and the hydroxide ion concentration in the solution is increased. This was also observed by other authors [50,53]. In addition, in most of the samples, the final pH was close to the inherent pH of Al-WTR. Because hydroxyl functional groups are essential in such reactions [55], higher Al hydroxide contents should lead to increased P adsorption capacity.

### 3.4. Correlation of P Adsorption with Chemical Properties of Al-WTRs

Statistical analysis was performed to correlate P adsorption capacities (Table 2) and chemical properties of Al-WTRs (Table 1) to identify a key property to estimate P adsorption potential. A correlation matrix was constructed to elucidate the relationship between P adsorption and various chemical properties (Table 3).

Maximum P adsorption capacity (q_m_) correlated strongly with oxalate-extractable Al content. It was clear that Al_ox_ controlled P adsorption on Al-WTRs. Hence, a simple linear model was constructed to determine how well this parameter alone could estimate q_m_. Results are shown in Table 4.

The F ratio for the simple linear regression is significant, indicating that the model successfully explains slightly more than 70% of the variability in adsorption capacity by evaluating Al_ox_ alone. The F ratio was well above 1, indicating that the model was a suitable predictor. The parameter estimate for Al_ox_ as a predictor of q_m_ is significant and positive, indicating that as Al_ox_ increases, an increase in q_m_ can be expected. The resultant simple linear expression to describe this relationship is demonstrated in Equation (7).

(7) $$q_{m}=0.1038\;[{\mathrm{Al}}_{\mathrm{ox}}\left(\mathrm{mg}/g\right)]+0.7900$$

Other variables may have also contributed to q_m_, so the analysis was expanded beyond a simple linear model. Data reduction techniques were considered to develop an efficient model. However, the correlation matrix did not indicate high correlations between variables, so principal component analysis was not used. A stepwise variable selection method was used to determine which variables may be significant. A backward selection method provided the best-fitting model. This regression method starts with a fully saturated model and sequentially eliminates variables from the model to reach an acceptable reduced model based on statistical contribution. The resultant model and equation are summarized in Table 5 and Equation (8), respectively. Variance inflation factors, a measure of multiple-variable collinearity, for included variables are all small, as shown in Table 5, indicating that multicollinearity is not a concern for the model. This can also be confirmed by examining the pairwise correlation matrix shown in Table 3 [56].

(8) $$q_{m}=0.1372\;[{\mathrm{Al}}_{\mathrm{ox}}\left(\mathrm{mg}/g\right)]-0.4162\;[{\mathrm{Fe}}_{\mathrm{ox}}\left(\mathrm{mg}/g\right)]-0.6455\left[S\left(\%\right)\right]+3.9124$$

According to the data presented in Table 5, using Al_ox_, Fe, and S contents of the Al-WTRs, a model could be built to estimate P adsorption capacity, capturing about 83% of data variability, providing an improvement relative to the simple model with one parameter. However, even in this model, the *p*-value for Al_ox_ was considerably lower than other parameters, meaning it was the most significant parameter.

The effect of interactions was also investigated, but none of the possible interactions improved the model. If the *p* values were desired to be less than 0.1, the model with Fe_ox_ and Al_ox_ could be considered a slight improvement (3% improvement in R square), but that was not favorable in this case.

## 4. Conclusions

In this study, we demonstrated that Al-WTRs from various locations in the US are nonhazardous and can be safely used for land applications for environmental remediation of P-enriched soils. All thirteen Al-WTRs collected from different parts of the US were capable of adsorbing P, regardless of their origin. However, their adsorption capacities varied depending on their chemical properties. Amorphous Al oxides/hydroxides contents in the Al-WTRs, measured by oxalate-extractable Al concentrations, could significantly predict their P adsorption capacity. A more robust model for prediction of P adsorption capacity of Al-WTRs was constructed by considering additional chemical properties, such as Fe_ox_ and S contents, which explained 83% of the variability in the adsorption data; however, Al_ox_ alone could explain more than 70% of the data variability. In summary, using Al-WTRs as adsorbent material for P is a sustainable remediation strategy, and their P adsorption efficiency can be evaluated before broadscale application by measuring a single chemical parameter, namely oxalate-extractable Al concentration. This new information provides stakeholders with a simple and inexpensive screening tool to determine whether a given residual could be reused for the remediation of P-enriched media.

## Figures and Tables

**Table 1:** Toxicity characteristic leaching procedure (TCLP) values and general chemical properties of Al-WTRs.

	Sample		NJ	MO	SD	NY	FL	OK	NE	KY	ME	CA	NC	MA	CT
		USEPA Limit													
**TCLP Values (mg/L)**	**As**	5	1.9	0.12	0.8	0.88	1.12	2.28	2.11	1.48	1.88	3.23	1.58	1.65	1.95
	**Ba**	100	1.44	2.25	0.46	2.02	0.27	3.25	1.43	2.39	0.1	1.88	BDL ^1^	BDL	BDL
	**Cd**	1	0.03	0.01	BDL	0.01	BDL	BDL	BDL	BDL	BDL	BDL	0.03	0.04	0.04
	**Cr**	5	0.02	0.01	0.01	BDL	0.07	0.04	0.05	0.04	0.01	0.04	0.02	0.18	0.25
	**Pb**	5	0.24	0.04	0.01	0.07	0.02	0.02	0.02	0.02	0.02	0.02	0.29	0.3	0.35
	**Hg**	0.2	BDL	BDL	0.01	BDL	BDL	BDL	0.01	BDL	BDL	BDL	BDL	BDL	BDL
	**Se**	2	BDL	BDL	BDL	BDL	BDL	BDL	BDL	BDL	BDL	BDL	BDL	BDL	BDL
	**Ag**	5	BDL	BDL	BDL	BDL	BDL	BDL	BDL	BDL	BDL	BDL	BDL	BDL	BDL
	**Cu**	NS ^2^	0.05	0.06	BDL	BDL	1.2	1.2	1.2	1.3	1.3	1.3	0	0.02	0.12
	**Ni**	NS	BDL	0.06	BDL	0.03	BDL	BDL	BDL	BDL	BDL	BDL	BDL	0.06	0.02
	**Zn**	NS	0.24	0.19	0.01	0.04	BDL	0.02	BDL	0.13	0.46	0.5	0.04	0.27	0.39
**EC (mS/cm)**			0.63	1.72	0.98	0.88	1.1	1.08	0.04	0.96	0.02	0.004	0.13	0.7	1.23
**pH**			6.08	7.3	9.79	7.47	8.47	9.4	7.75	7.39	6.36	7.05	6.03	7.27	6.71
**OM (%)**			14.31	0.45	2.32	1.17	0.11	0.29	0.16	3.66	7.03	5.84	3.37	1.8	2.11
**C (%)**			8.24	3.91	10.64	6.26	12.29	9.89	11.33	12.04	17.15	10.03	3.81	7.61	11.7
**N (%)**			0.78	0.47	0.21	0.64	0.12	0.17	0.12	0.91	0.96	1.33	0.74	0.8	1.04
**S (%)**			8.13	1.68	0.33	4.14	0.35	0.32	0.33	5.45	13.43	9.6	0.49	0.74	1.65
**Alox (mg/g)**			143.75	15.06	1.14	71.9	0.27	1.1	0.9	72.84	50.35	139.66	76.01	151.52	166.54
**Feox (mg/g)**			6.59	5.67	0.99	6.68	0.48	9.82	1.34	3.87	7.34	2.49	21.74	22.18	7.46
**Pox (mg/g)**			1.96	0.49	0.3	0.45	0.01	0.12	0.08	0.66	0.14	1.17	0.35	0.21	0.74
**Total P (mg/g)**			2.76	0.7	0.4	0.9	0.04	0.15	0.96	1.92	0.51	2.5	2.4	0.8	0.86
**PSI (%)**			1.16	2.4	16.12	0.52	2.59	1.77	4.31	0.77	0.23	0.73	0.35	0.11	0.38

^1^ Below detection limit; ^2^ not specified.

**Table 2:** Maximum adsorption capacity of Al-WTRs.

Sample (State)	NJ	MO	SD	NY	FL	OK	NE	KY	ME	CA	NC	MA	CT
**q_m_ (mg/g)**	12.73	3.50	2.44	6.55	1.20	3.67	2.82	6.72	1.47	15.95	5.35	11.68	28.72

**Table 3:** Correlation matrix.

	q_m_ (mg/g)	Al_ox_ (mg/g)	Fe_ox_ (mg/g)	P_ox_ (mg/g)	Total Al (mg/g)	Total Fe (mg/g)	Total P (mg/g)	PSI	pH	OM (%)	C (%)	N (%)	S (%)
**q_m_ (mg/g)**	1.0000												
**Al_ox_ (mg/g)**	**0.8521**	1.0000											
**Fe_ox_ (mg/g)**	0.1404	0.3998	1.0000										
**Pox (mg/g)**	0.5363	0.6395	−0.1123	1.0000									
**Total Al (mg/g)**	0.6022	0.8511	0.5893	0.4452	1.0000								
**Total Fe (mg/g)**	−0.3701	−0.4834	0.2266	−0.4191	−0.3781	1.0000							
**Total P (mg/g)**	0.3520	0.5846	0.1840	0.7698	0.6238	−0.1739	1.0000						
**PSI**	−0.3243	−0.4913	−0.4111	−0.1887	−0.5782	0.0415	−0.3159	1.0000					
**pH**	−0.4045	−0.6521	−0.3931	−0.4807	−0.8257	0.3027	−0.6512	0.6881	1.0000				
**OM (%)**	0.2422	0.5104	0.0074	0.8189	0.4975	−0.3804	0.6754	−0.1718	−0.5628	1.0000			
**C (%)**	−0.0227	−0.1063	−0.4680	−0.1752	−0.1380	−0.2008	−0.3125	0.0898	0.1443	0.1299	1.0000		
**N (%)**	0.6494	0.8293	0.2404	0.5396	0.8986	−0.5541	0.6100	−0.5212	−0.7085	0.5108	0.0710	1.0000	
**S (%)**	0.0969	0.3421	−0.1883	0.4676	0.4749	−0.4627	0.3908	−0.3309	−0.5141	0.7141	0.4462	0.6653	1.0000

**Table 4:** Simple linear model between q_m_ and Al_ox_.

**Source**	**DF**	**Sum of Squares**	**Mean Square**	**F Ratio**
Model	1	526.369	526.369	29.167
Error	11	198.516	18.047	**Prob > F**
C. Total	12	724.886		0.0002 *
**Term**	**Estimate**	**Std Error**	**t Ratio**	**Prob > |t|**
Intercept	0.790	1.768	0.45	0.664
Al_ox_ (mg/g)	0.104	0.019	5.40	0.0002 *
RSquare Adj	0.701			

* Variable is significant.

**Table 5:** Multivariate model between q_m_ and chemical characteristics of Al-WTRs.

**Source**	**DF**	**Sum of Squares**	**Mean Square**	**F Ratio**	
Model	**3**	631.932	210.644	20.395	
Error	9	92.954	10.328	**Prob > F**	
C. Total	12	724.886		0.0002 *	
**Term**	**Estimate**	**Std Error**	**t Ratio**	**Prob > |t|**	**VIF**
Intercept	3.912	1.658	2.36	0.0427 *	
Al_ox_ (mg/g)	0.137	0.018	7.66	<0.0001 *	1.5164
Fe_ox_ (mg/g)	−0.416	0.155	−2.68	0.0250 *	1.3881
S (%)	−0.645	0.246	−2.62	0.0278 *	1.3208
RSquare Adj	0.829				

* Variable is significant.

## Data Availability

The data presented in this study are available in the Supplementary Materials.

## Supplementary Materials

The following supporting information can be downloaded at: https://www.mdpi.com/article/10.3390/molecules27217194/s1, Table S1: Total metal(loid) concentrations of Al-WTRs; Table S2: Synthetic precipitation leaching procedure (SPLP) values of Al-WTRs; Figure S1: Final pH after adsorption experiment.

## Sample Availability

Samples of the compounds are not available from the authors.
