# Supplementary material for: Correlation of Phosphorus Adsorption with Chemical Properties of Aluminum-Based Drinking Water Treatment Residuals Collected from Various Parts of the United States
Source: Molecules. 2022 Oct 24;27(21):7194. doi: 10.3390/molecules27217194 (PMC9656969; doi:10.3390/molecules27217194)
Supplement: Supplementary file 1 [file molecules-27-07194-s001.zip › molecules-1976657-supplementary(1).pdf]

Supplementary Data

Correlation of Phosphorus Adsorption with Chemical Properties of Aluminum-Based Drinking Water Treatment Residuals Collected from Various Parts of the United States

Roxana Rahmati, Virinder Sidhu, Rosita Nunez, Rupali Datta, Dibyendu Sarkar

Table S1. Total metal(loid) concentrations of Al-WTRs

	As	Pb	Cd	Cr	Cu	Ni	Zn	Al	Fe
			(mg/kg)					(mg/g)	
**NJ**	19.9	17.4	0.43	22.1	59	15.7	101	158.49	14.07
**MO**	76.3	14	0.55	36.5	35.1	32.6	84.6	66.97	37.80
**SD**	4.67	2.99	0.06	0.42	5.60	4.81	19.7	3.60	27.17
**NY**	27.5	17.8	0.61	26	33.1	30.7	90.6	99.69	28.08
**FL**	7.65	3.49	0.05	BDL	2.0	BDL[1]	7.37	0.62	8.20
**OK**	3.37	4.52	0.19	9.66	7.91	9.22	25.6	11.55	145.04
**NE**	20.3	2.27	0.15	BDL	6.41	3.66	2.91	2.22	125.33
**KY**	17.3	5.65	0.24	14.1	75.8	16.6	86.8	112.30	12.92
**ME**	7.15	8.89	0.29	11.5	106	4.74	50.3	174.06	18.16
**CA**	111	3.73	0.16	31.2	39.5	17.4	68.8	203.32	8.02
**NC**	4.75	14.8	0.55	44.4	37.9	20.5	103	222.98	88.95
**MA**	28.5	7.45	1.99	125	94.5	29	82.5	193.04	30.96
**CT**	6.49	3.72	0.17	162	116	4.81	60.1	193.63	7.97

[1]Below detection limit

Table S2. Synthetic Precipitation Leaching Procedure (SPLP) values of Al-WTRs (all units are mg/L)

	As	Ba	Cd	Cr	Pb	Hg	Se	Ag	Cu	Ni	Zn
**USEPA limit**	5	100	1	5	5	0.2	1	5	NS[1]	NS	NS
**NJ**	BDL	0.05	BDL	BDL	BDL	BDL	BDL	BDL	BDL	BDL	BDL
**MO**	BDL	0.03	BDL	BDL	BDL	BDL	BDL	BDL	BDL	BDL	BDL
**SD**	BDL	BDL	BDL	BDL	BDL	BDL	BDL	BDL	BDL	BDL	BDL
**NY**	BDL	0.11	BDL	BDL	BDL	BDL	BDL	BDL	BDL	BDL	BDL
**FL**	BDL	0.05	BDL	BDL	BDL	0.01	BDL	BDL	BDL	BDL	BDL
**OK**	0.02	BDL	BDL	BDL	BDL	BDL	BDL	BDL	0.03	BDL	BDL
**NE**	BDL	0.04	BDL	BDL	BDL	BDL	BDL	BDL	0.06	BDL	BDL
**KY**	0.02	BDL	BDL	BDL	BDL	BDL	BDL	BDL	0.01	BDL	BDL
**ME**	0.07	0.04	BDL	BDL	BDL	BDL	BDL	BDL	0.34	BDL	BDL
**CA**	0.02	BDL	BDL	BDL	BDL	BDL	BDL	BDL	0.05	BDL	0.02
**NC**	0.5	0.07	BDL	BDL	BDL	BDL	BDL	BDL	0.34	BDL	BDL
**MA**	BDL	BDL	BDL	BDL	BDL	BDL	BDL	BDL	BDL	BDL	BDL
**CT**	BDL	BDL	BDL	BDL	BDL	BDL	BDL	BDL	BDL	BDL	BDL

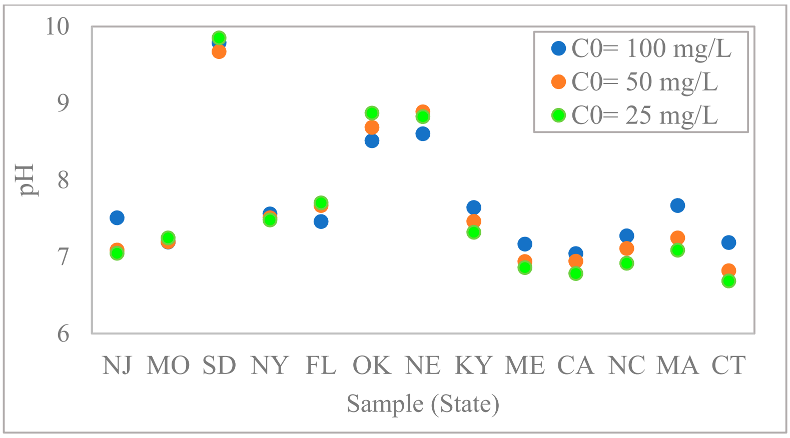

Figure S1. Final pH after adsorption experiment
